# Design of Efficient Full Adder in Quantum-Dot Cellular Automata

**DOI:** 10.1155/2013/250802

**Published:** 2013-06-05

**Authors:** Bibhash Sen, Ayush Rajoria, Biplab K. Sikdar

**Affiliations:** ^1^Department of Computer Science and Engineering, National Institute of Technology, Durgapur, India; ^2^Department of Computer Science and Technology, Bengal Engineering and Science University, Shibpur, India

## Abstract

Further downscaling of CMOS technology becomes challenging as it faces limitation of feature size reduction. Quantum-dot cellular automata (QCA), a potential alternative to CMOS, promises efficient digital design at nanoscale. Investigations on the reduction of QCA primitives (majority gates and inverters) for various adders are limited, and very few designs exist for reference. As a result, design of adders under QCA framework is gaining its importance in recent research. This work targets developing multi-layered full adder architecture in QCA framework based on five-input majority gate proposed here. A minimum clock zone (2 clock) with high compaction (0.01 **μ**m^2^) for a full adder around QCA is achieved. Further, the usefulness of such design is established with the synthesis of high-level logic. Experimental results illustrate the significant improvements in design level in terms of circuit area, cell count, and clock compared to that of conventional design approaches.

## 1. Introduction

Current CMOS-based architecture is on the verge of reaching the limit of feature size reduction. Its high power consumption also prevents the energy-efficient realization of complex logic circuits at nanoscale. Also, downsizing of CMOS circuitry does not necessarily produce corresponding gains in device density [1]. The alternatives to conventional CMOS technology, for attaining high computational power and compact design density, are therefore being investigated [2, 3]. Quantum-dot cellular automata (QCA) is introduced to create nanoscale devices with high compaction density [4], capable of performing computation at very high switching speed [5]. The small QCA cells cause QCA interconnect to shrink, thereby increasing device density. Recent research explores that QCA (magnetic QCA) can be operational at room temperature [6].

QCA accomplishes logical operations and moves data through pure Coulombic interactions rather than transport of charge between the cells. Conventional binary information is represented by the configuration of electron of QCA cell. The fundamental QCA logic primitives are the three-input majority gate, wire, and inverter [7]. Since the majority gate is not functionally complete, the majority gate with inverter, called MI, is used to realize the different QCA designs. Also, cell layout and timing constraints are inevitable steps in mapping a digital design to the majority of logic-based QCA circuits cells. However, the wide acceptance of QCA-based designs demands introduction of efficient design methodologies to address the issue of its susceptibility to high error rate at nanoscale.

Wire crossings play a key role in systematic logic design [8, 9]. Also, wire crossing poses a bigger barrier than wire length in QCA architecture [10]. In the classic binary QCAs, wire cross is realized either considering rotated QCA cells in a wire (coplanar wire crossing) or with multilayer crossing. In coplanar crossings, each section is loosely coupled to the other section of horizontal wire. Such a floating structure is susceptible to random external effects. Furthermore, unlike present CMOS integrated circuits, where metal layers cannot perform any intelligent functions but to connect discontinuous sections of a circuit, an extra layer in the multilayered QCA architecture can be used as the active component of the circuit [11, 12].

Although the multilayer approach proves to be more robust [13], the majority of designs employ the coplanar one due to its simplicity; another approach exploits the pipelined nature of QCA and uses parallel-to-serial converters and a specialized clocking scheme to design a coplanar crossbar network [8]. In coplanar approach, the layout area of complex circuits involving considerable number of complex Boolean functions becomes too huge to be practically not acceptable in nanoscale arena. This problem of large effective circuit area (mostly wire crossing and large number of logic gates) can be reduced by the introduction of multilayer architecture. Although a two-layer approach is explored for QCA ternary logic [14], multilayer approach for classic QCA (binary) is still not explored.

Recently, few QCA designs for a cost-effective adder were investigated in [15–17]. However, all of these investigations were limited mostly to coplanner QCA layout with few exception with multilayer wire crossing only. QCA processing of intercell interaction is also applicable for interlayer interaction. In a multilayer case, two cells are closest when placed directly one over the other, that is, on the same location but on separate layers. To date, multilayered designs have mostly used the concept for wire crossing only.

This motivates us to design an efficient multilayer QCA architecture with proper analysis of the effect of layer spacing and radius of effect of different QCA cell sizes. The novelty of this paper lies in realizing the design issues associated with multilayered QCA architecture. Due to the unique clocking scheme (four-phase clocking zone) used in QCA, minimizing the clock zone becomes a very critical issue for realising cost-effective multilayer design. Besides synchronized multilayer wire crossings, our current research is devoted to the study of multilayer approach that consumes fewer clock cycles as well. However, in this paper, through the design of a full adder, we have shown the utility of multilayered approach in synthesis of logic circuits. A scheme for modelling digital devices around five-input majority gate followed by a more feasible full adder unit has been framed with the target to achieve high device density in QCA designs. The major contributions of this work around multilayer architecture can be summarized as follows.

- Realization of most compact multilayered structure of 5-input majority voter.
- Design of cost-effective full adder based on proposed 5-input majority gate.
- Use of different layers as active circuit component followed by robust wire crossing.
- Manufacturing defects like cell displacement, deposition, and redundancy in cell position are also examined.
- Finally, synthesis of high-level complex logic circuit using proposed full adder is also investigated.

Simulations using QCA Designer [18] supports all the results presented.

This paper is organised as follows.  Section 2 deals with preliminaries including a brief overview of QCA technology. Related works on this QCA architecture are explored in Section 2.2. Multilayer design of 5-input majority gate followed by a full adder is presented in Section 3. In Section 3.1.1, the defect tolerance of the proposed QCA adder is analysed. In Section 3.2, different QCA circuits such as 4-bit, 8-bit ripple carry adders are synthesized with this full adder. Discussion and conclusion are given in Section 4.

## 2. Preliminaries

**Figure 1:**
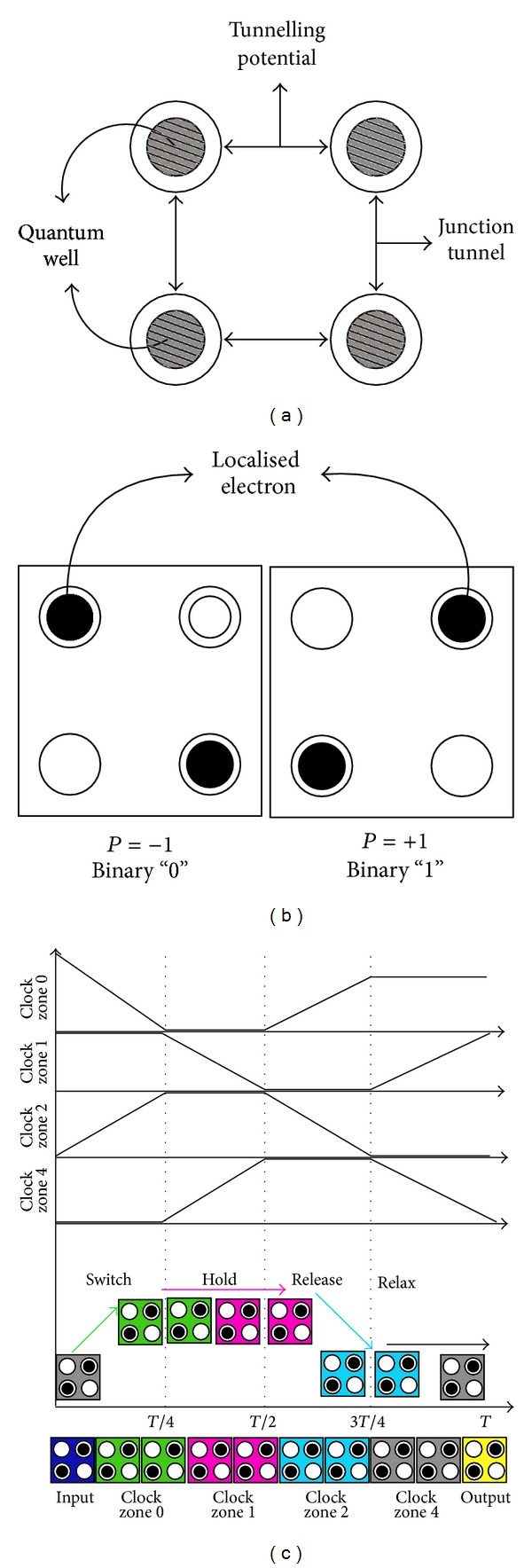
(a) QCA cell and (b) QCA cell with two different polarization. (c) Clocking.

In QCA-based design, a single device (QCA-cell) is used for the construction of all components of an entire circuit (computational elements and wires). The schematic diagram of a four-dot QCA cell is shown in Figure 1. The cell consists of four quantum dots positioned at the corners of a square and contains two free electrons [4]. A quantum dot is a region where an electron is quantum-mechanically confined (Figure 1(a)). Coulombic repulsion will cause classical models of the electrons to occupy only the corners of the QCA cell, resulting either in polarization *P* = −1 (logic 0) or in *P* = +1 (logic 1) as shown in Figure 1(b).

Timing/synchronization in QCA is accomplished by the cascaded clocking of four distinct and periodic phases as shown in Figure 1(c) [19]. In the first (switch) phase, the tunnelling barrier between two dots of a QCA cell starts to rise. This is the phase during which computation takes place. The second (hold) phase is reached when the tunnelling barriers are high enough to prevent electrons from tunnelling. In the third (release) phase, barrier falls from high to low. The final phase (relax) ensures there is no interdot barrier and the cell remains unpolarized. Each cell has to pass either of these clocking zones.

### 2.1. QCA Logic Gate

**Figure 2:**
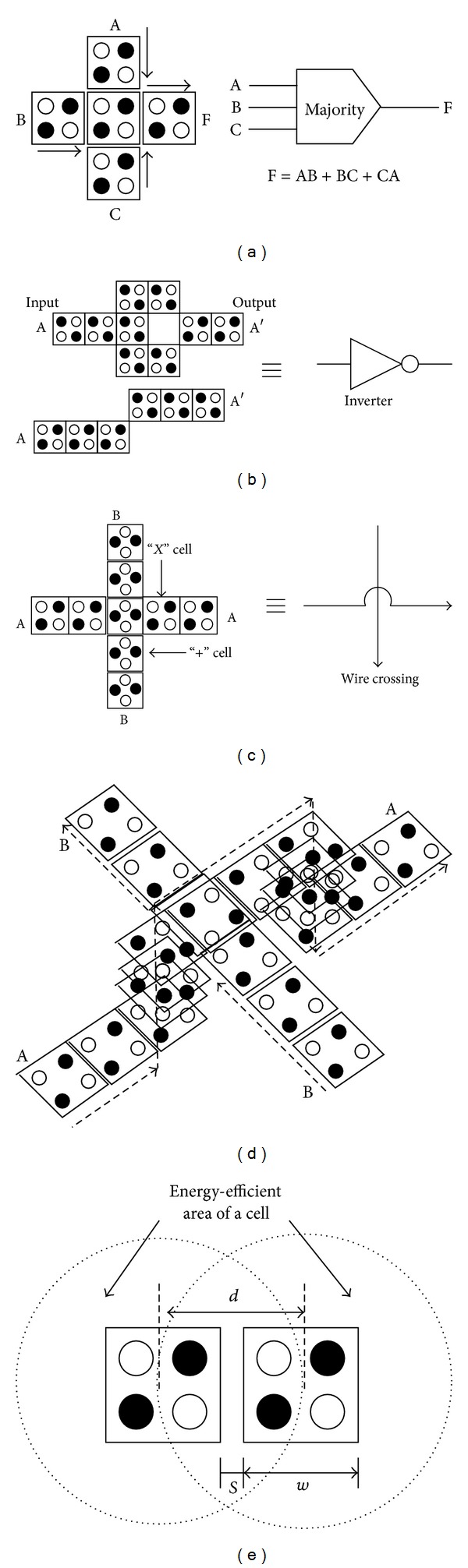
(a) Majority voter, (b) inverter, (c) coplanar wire crossing, (d) multilayer wire crossing, and (e) area under induced effect of majority cell.

The basic structure realized with QCA is the 3-input majority gate, MV(A, B, C) = Maj(A, B, C) = AB + BC + CA (Figure 2(a)). The majority gate can also function as a 2-input AND or a 2-input OR by fixing one of the three input cells to *P* = −1 or *P* = 1, respectively. Inversion can be done within the wire by slightly off-centering the wire. Thus, it is realized in two different orientations as shown in Figure 2(b). In [20], the constraints imposed by the radius of effect of each cell is described which defines the distance *d* that can affect the operation of certain structures in QCA array. That is, two in-line QCA cells interact if

(1) $$\begin{array}{c} d=d_{N}=w+s, \end{array}$$

where *w* is the width (and height) of (square) cell and *s* is the measure of separation between two consecutive cells (Figure 2(e)). The other different radius of effect for nearest diagonal/next to neighbour is described in [20].

In QCA, two kinds of QCA wire crossings are possible to be found, like coplanar (Figure 2(c)) and multilayer (Figure 2(d)). Coplanar wire crossing in QCA requires two different orientations, a 90° (×−cell) and a 45° (+−cell) structure whereas multilayer wire crossing has no such strict orientation limit. A multilayer crossover is quite straightforward from the design perspective and the signal connection is steadier. The probability of undesirable crosstalk between any two crossing lines can be avoided by introducing multilayer wire crossing. Also, in a coplanar crossing, there is a possibility of a loose binding of the signal which causes a discontinuity of the signal propagation, and there is the possibility of back propagation from the far side constant input. So putting enough clock zones between the regular cells across the rotated cells is required. In this paper, all the designs are established mostly on multilayer wire crossing.

### 2.2. Related Work

The first QCA full adder design was presented in [7]. This design is constructed using five three-input majority gates and three inverters. A simpler QCA full adder was presented in [21]. This full adder is composed of three three-input majority gates and two inverters. Using this design, different layouts for a QCA full adder have been presented to date [15].

Recently, a novel QCA full adder design was introduced in [22]. This design is composed of one three-input majority gate, one inverter, and a new kind of majority gates: a five-input voter. This study also presents an unconventional form of three-dimensional (3D) QCA cells. Based on the presented design in [22], different QCA full adders have been introduced [23]. However, owing to some problems in simulation and physical implementation of 3D QCA cells in comparison to the classic ones, this design seemed not to be appropriate, at least at present [17]. As a consequence, it cannot be assured if such an implementation possiblly can drastically reduce cell count, area, and clock cycles.

A few recent research considered multilayer architecture only for its advantages in wire crossing [16, 17, 24]. In [17], a new five-input majority gate (5-MV) is proposed and a new full adder based on that 5-MV is synthesized. So far, the idea of treating each layer as active layer for function realisation unlike CMOS has not been investigated (which is of primary interest to us in this paper).

## 3. Design of Efficient Full Adder

The most important mathematical operation is addition. Other operations such as subtraction, multiplication, and division are usually implemented by adders. So an efficient adder can be of great assistance in designing arithmetic circuits. Recently, it is shown that 1-bit full adder can be realized with 3 majority gates and one inverter [24]. The total circuit delay is of 1 clock a (4 clock zones) for generating the outputs.

**Figure 3:**
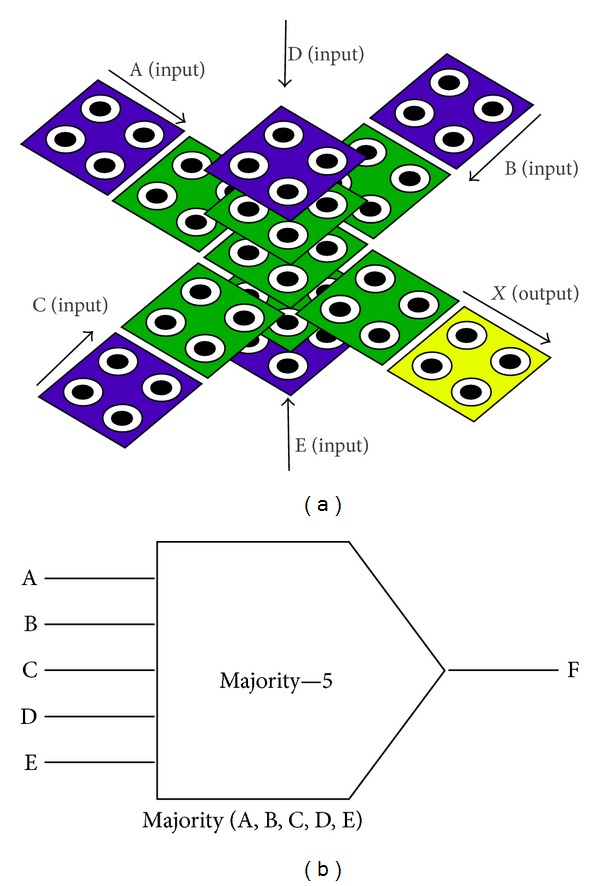
Block diagram of five-input majority gate.

**Figure 4:**
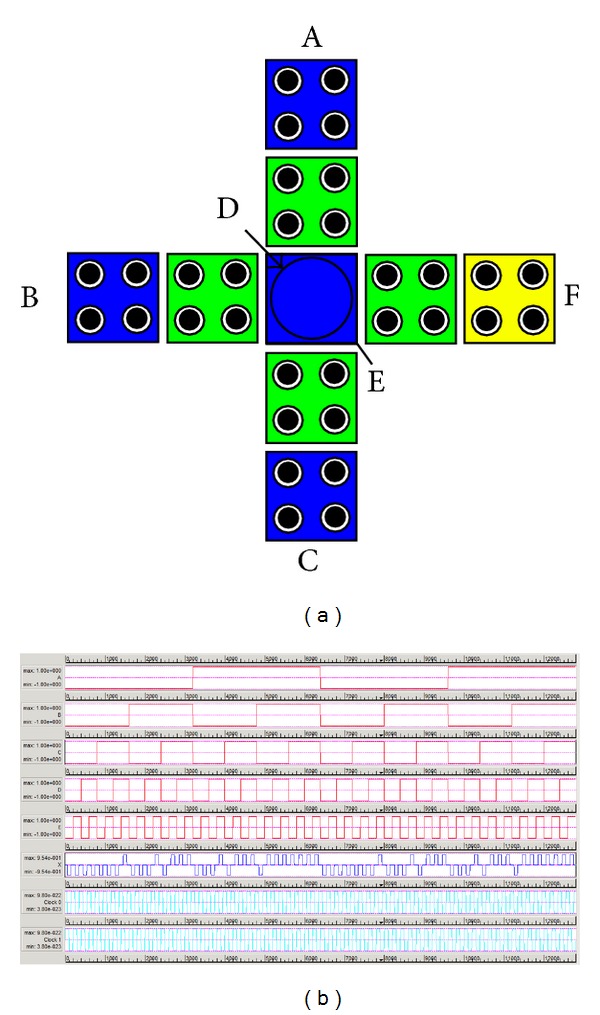
QCA cell layout and its simulation result of five-input majority gate.

In order to minimize the number of majority gates and inverters, a multilayer design using *5-input majority* gate is proposed here (Figure 3). A five-input majority gate is a Boolean gate whose output is 1 only if 3 or more of its inputs is 1. The Boolean function of a five-input majority gate is F(A, B, C, D, E) = ABC + ABD + ABE + ACD + ACE + ADE + BCD + BCE + BDE + CDE. A 3-input majority has been implemented using only one design to date. However, a 5-input majority gate can be implemented using various designs. The block diagram of our proposed 5-input majority gate is as shown in Figure 3(b). QCA cell layout and its simulation of 5 input majority voter is shown in Figure 4. The comparative analysis establishes that this structure is more compact than the other reported 5-input majority gate designs (Table 1). This gate covers 0.0096 *μ*m^2^ and uses the least clock zone required.

*Multilayer Architecture*. Layer 1 has one input (E), layer 3 has three inputs (A, B, C), and layer 5 has one input (D). The desired output is obtained from layer 3. In this design, the output is not surrounded by the other cells, and therefore, it can easily be accessed. In other words, this structure does not need any wire crossover to transmit the output signal. Hence, the output can be easily fed into the input of the other QCA circuits. The use of five layers to implement a 5-input majority gate using multilayer approach is necessary because the input signals get inverted as we move across layers. Though, it can be made in three layers, also. In that case, upper-layer cell should be placed in diagonal position of the lower cell instead of top of it directly. Our proposed design uses only *one clock* zone, and hence there is *no delay* between the input and the output.

**Lemma 1**

The minimum number of clock zones required to realize a 1-bit full adder using 5-input majority gate is two.

**Proof**

The Boolean function for the sum and carry out bit for a 1-bit full adder is given by SUM = A ⊕ B ⊕ C, CARRY = AB + BC + CA. The above Boolean function is implemented using 3-input majority and 5-input majority gates as follows: $\text{SUM}=\text{M}5(\text{A},\text{B},\text{C},\bar{\text{CARRY}},\bar{\text{CARRY}})$, CARRY = M3(A, B, C). The carry bit is generated using a traditional 3-input majority gate in the first layer and is directly transmitted to the output and it requires at least one clock zone. Then, this carry bit is propagated upwards using multilayer crossover scheme by placing a cell in the second layer diagonally across the output carry cell. Thus, the output carry signal appears as CARRY in the second layer. This CARRY signal is eventually fed into the 5-input majority gate in the third layer. Cells are also stacked over the input cells A, B, and C of the first layer to propagate the input signals to third layer. The input signals A, B, and C so obtained in the third layer using multilayer concept are also fed as input to the 5-input majority gate. The output of the 5-input majority gate is the required sum bit. Since, the output of the 3-input majority gate which is present in the third layer is being fed into the 5-input majority gate which is present in the third layer, an additional one clock cycle is mandatory for stable output. Therefore, at least two clocks are necessary to get a stable output for the design of a 1-bit full adder using five input majority gate.

**Figure 5:**
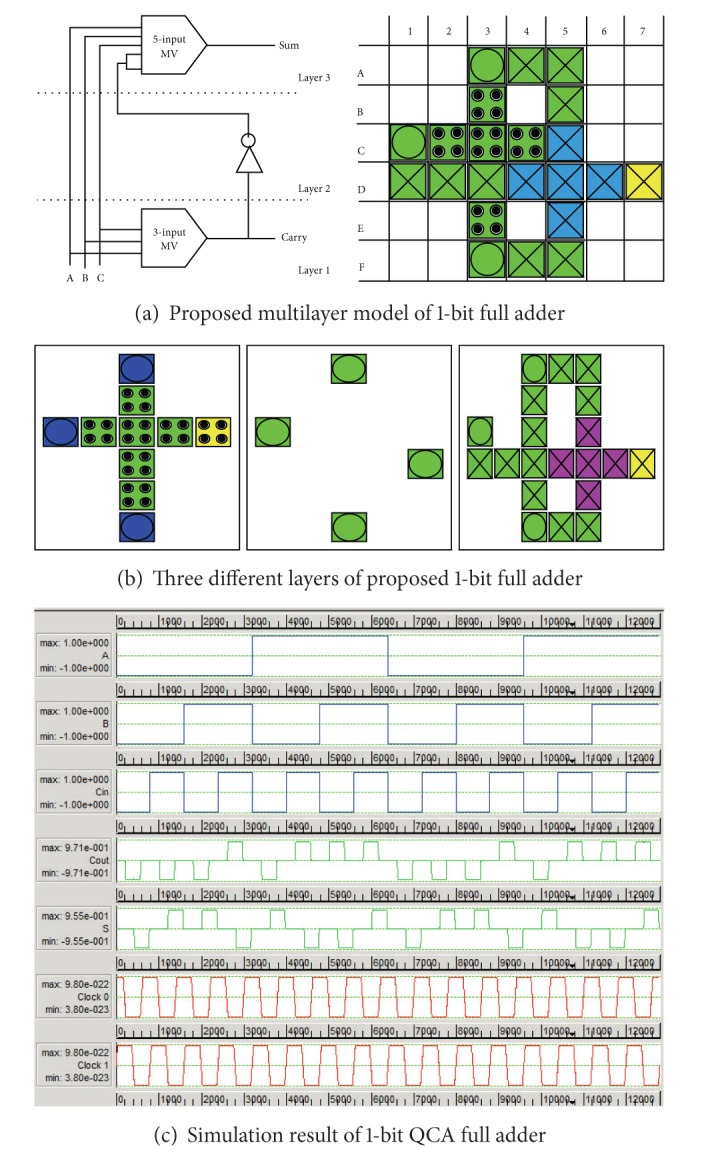
QCA implementation of the proposed full adder and its simulation result.

The multilayer architecture of full adder is designed using two majority gates (one 5-input gate and one 3-input majority) and two clock zone (Figure 5(a)). Corresponding cell layout is given in Figure 5(b) and its simulation result is shown in Figure 5(c). No inverter is required as inversion can take place within multilayer itself. So, multilayering also reduces the number of logic gate and propagation delay as required.

This design leads to around 39.22% improvement in terms of number of cells used and 48.15% improvement in terms of area in comparison to the existing multilayer QCA full adder design constructed using three-input majority and five-input majority gates in [17] (Table 2).

### 3.1. Characterization of Fault Tolerance under Different QCA Defects

In this section, different types of QCA defects are investigated for the proposed full adder. Characterization of these defects explores the robustness and the fault tolerance limit with respect to manufacturing process variations. According to [25], in the present stage of QCA manufacturing, defects are possible in both the chemical synthesis phase, in which the individual cells (molecules) are manufactured, and the deposition phase, in which cells are placed in a specific location in the surface. Manufacturing defects during chemical synthesis may cause a cell to have missing or extra dots or/and electrons. However, defects are much likely to occur during deposition than chemical synthesis (which will result in cell misplacement). These defects are mainly categorized into three parts.

**Figure 6:**
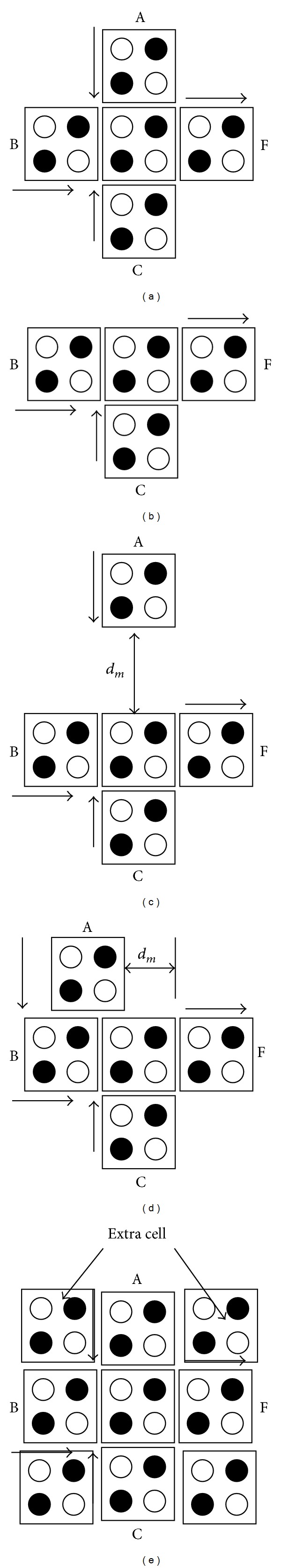
(a) Fault-free majority gate, (b) cell omission, (c) cell displacement, (d) cell misalignment, and (e) extra/additional cell.

- Cell displacement and misalignment: the defective cell is displaced from its original direction (Figures 6(c) and 6(d)).
- Cell omission/missing: a particular cell is missing or remains undeposited in the original (defect-free) configuration (Figure 6(b)).
- Additional cell deposition: an additional cell is deposited on the substrate (Figure 6(e)). This extra cell is erroneously deposited along the device perimeter (adjacency boundary) of the original (defect-free) configuration.

All the identified defects in QCA tiles are shown in (Figure 6).

These defects in different parts of full adder, including straight wires, corners, majority voters, inverters, and crossovers, have been considered and simulated as reported in the following sections.

#### 3.1.1. Cell Displacement Defect

Cell displacement errors are quite frequent during fabrication of a design.  Table 3 reports the displacement tolerance value of each cell to generate correct output of the 3-layer QCA full adder. The values are obtained for the 3-layer with 18 × 18 nm^2^ cell technology. The cells C3, D5 (the device cells of the majority gates), and E5 are highly vulnerable to such displacements only (see Figure 5(a)), whereas the other cells (not integrated with the inner part of the design) are more tolerant to such displacements.

#### 3.1.2. Cell Omission/Missing Defect

**Figure 7:**
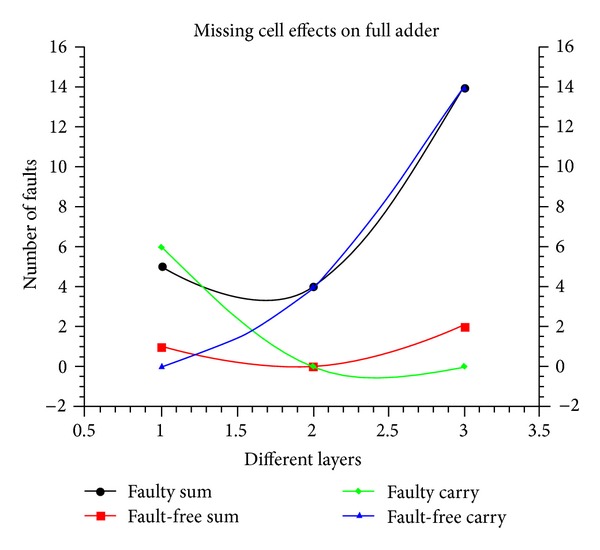
Missing cell defect.

The behaviour of the full adder under single missing cell defects is reported in Figure 7. The values 1, 2, and 3 along *x*-axis indicate the layer, and the total faults occurred in SUM and CARRY outputs of the full adder are captured along *y*-axis. In layer 3, the carry output is mostly fault free compared with other two layers. Simulation results show that cell omission defect on the crossover and vertical cell affect the sum functionality of the circuit.

#### 3.1.3. Additional Cell Defect

**Figure 8:**
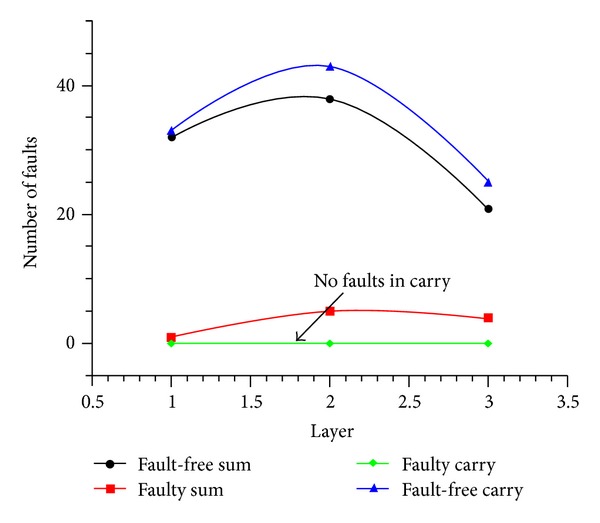
Additional cell defect.

The behaviour of the full adder under single additional cell defects is reported in Figure 8. The values 1, 2, and 3 along *x*-axis indicate the layer, and the total faults occurred in SUM and CARRY outputs of the full adder are captured along *y*-axis. Simulation results show that additional cell deposition defect on each layer does not affect the carry functionality of the circuit. From Figure 8, it is evident that the proposed full adder is more fault tolerant under extra cell deposition.

### 3.2. Logic Synthesis Using the Proposed Full Adder

**Figure 9:**
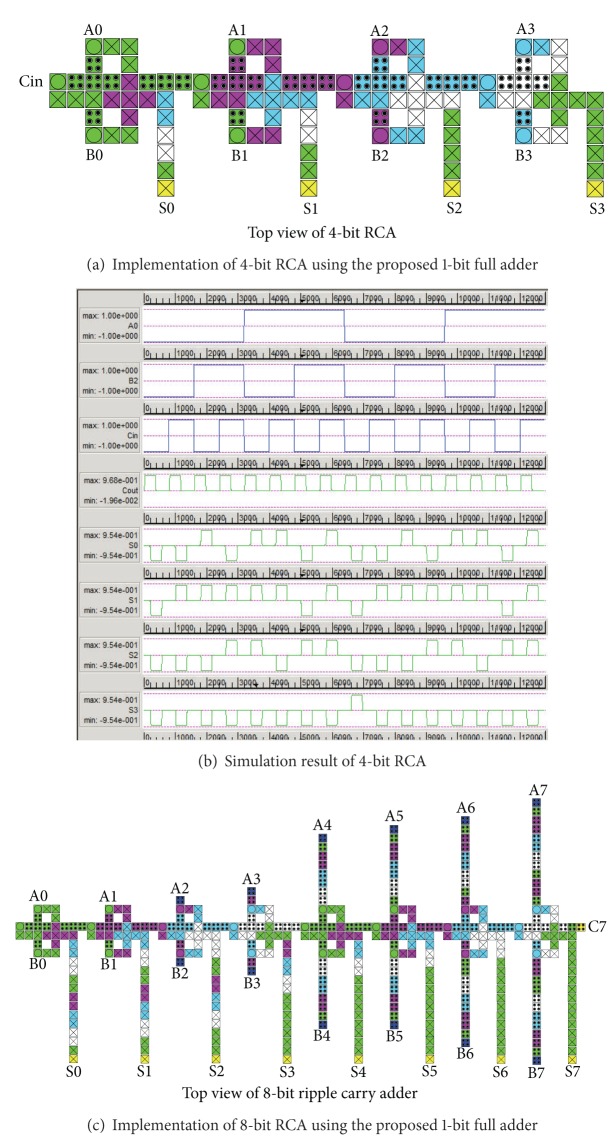
QCA implementation of different ripple carry adder (RCA) circuits with the proposed 1-bit full adder.

Design capability of the proposed model is further analysed by implementing 4-bit and 8-bit RCA (Figure 9). In [17], a detailed comparison between the QCA full adder proposed in [17] and the previous designs is reported. To make it comprehensible, Table 4 demonstrates a detailed comparison between the proposed QCA full adders and the best previous design [17]. Based on the results in Table 4, it is clear that the new ripple carry adders lead to significant improvements in terms of area, delay, and complexity in comparison to the best previous designs. Design complexity, delay, and area consumption of QCA circuits are obtained by QCA designer [18].

## 4. Conclusion

In this work, a multilayer architecture of a full adder around QCA (quantum-dot cellular automata) is introduced considering its primitives (majority voter). This design has a Simple layered structure and is constructed using a new five-input majority gate proposed here. The resulting design takes only two clocking zones (lowest) covering an area of 0.01 *μ*m^2^ which can never be achieved with existing coplanar designs because of their layout and timing constraints. The usefulness of the proposed design is further analysed with the implementation of ripple carry adders of different word sizes (i.e., 4 and 8) which lead to significant improvements in terms of area, delay, and complexity in comparison to the best previous designs. The fundamental issues related to multilayer architecture are addressed on all levels of design. Its robustness and signal steadiness issues are further analysed with different cell deposition defect.

Our current research is devoted to the study of active multilayer circuit with synchronized multilayer wire crossings that consume fewer clock cycles, which we find to be one of the more promising approaches for QCA design in general. Though, the clocking structure beneath the QCA cell layer is also very important and nontrivial research issue.

## Figures and Tables

**Table 1:** Comparison of five-input majority gate.

Design	Layer	Clock no.	Cell no.	Area
In [22]	Undefined	0.50	18	7 × 6
In [17]	Coplanar	0.50	18	6 × 7
Proposed	Multilayer	0.25	13	5 × 5 × 5

**Table 2:** Comparison of the recent 1-bit full adder.

Design	Cell count	Area *μ*m^2^	Clock no. cycle	MV gate no.	Inv gate no.
In [16]	135	0.14	1.25	3 (3 MV)	2
In [15]	108	0.10	1	3 (3 MV)	2
In [17], type I	79	0.05	1.25	2 (3 MV) 1 (5 MV)	2
In [17], type II	51	0.03	0.75	1 (3 MV) 1 (5 MV)	2
This work	31	0.01	0.50	1 (3 MV) 1 (5 MV)	0

**Table 3:** Permissible cell displacement of the proposed full adder.

Cell	Left←	Right→	Up↑	Down↓
Layer 1				
A3	5.7	5.8	3.6	—
B3	4	3.4	—	—
C1	4.7	—	6.1	6.1
C2	—	—	5	4.3
C3	—	—	—	—
C4	—	—	4.9	4.9
D3	4.3	3.6	—	—
E3	5.8	5.9	—	—
F3	7.4	7.2	7	—
Layer 2				
A3	6.1	4.3	6.1	5.8
C1	6	5.5	6	4.3
D5	2.5	2	2.7	1.9
F3	6.2	4.6	5.5	6.2
Layer 3				
A3	3.4	—	4.5	4.5
A4	—	—	∞	5.6
A5	—	5.6	5.5	—
B5	5.1	6.2	—	—
C1	4.7	3.4	3.2	—
C5	4.7	3.8	—	—
D1	4	—	—	3.5
D2	—	—	5.9	22.9
D3	—	—	∞	18.7
D4	—	—	6.1	5.8
D5	—	—	4.2	4.4
E5	2.9	2.9	—	—
F3	3.8	—	4.7	4.7
F4	—	—	6.9	7.2
F5	—	3.8	—	2.2

**Table 4:** Comparison of recent ripple carry adder (RCA).

Design	Cell count	Area *μ*m^2^	Clock cycle
4-bit			
In [17], type I	558	0.85	1.25
In [17], type II	308	0.29	2
Proposed here	153	0.11	1
8-bit			
In [17], type I	1528	2.93	9
In [17], type II	695	0.79	3
Proposed here	466	0.77	2.25
